# Anton Ludovico Antinori’s rheumatological ailments: established facts or overinterpretation?

**DOI:** 10.1093/rheumatology/kead170

**Published:** 2023-04-17

**Authors:** Raffaella Bianucci, Simon Donell, Francesco M Galassi

**Affiliations:** Legal Medicine Section, Department of Public and Pediatric Sciences, University of Turin, Turin, Italy; Department of Cultures and Societies, University of Palermo, Palermo, Italy; Ronin Institute, Montclair, NJ, USA; Norwich Medical School, University of East Anglia, Norwich, UK; Department of Anthropology, Faculty of Biology and Environmental Protection, University of Łódź, Łódź, Poland

Rheumatology key message

- Anton Ludovico Antinori’s disease cannot be said to be giant cell arteritis and polymyalgia rheumatica for certain, nor is this case a superior source of evidence for the occurrence of these diseases in the past.

Dear Editor, We have read with interest the note on the potential PMR and GCA of Anton Ludovico Antinori (1704–1778) by L. Ventura and A. Foscati [1] and the discussion of the differential diagnosis that highlights the educational elements of discussing historical figures from the past. The prospect of an exhumation of the remains with an associated palaeogenetic study [1] makes their retrospective diagnosis relevant to the palaeopathological debate, since many of the graves of historical individuals suggested to have suffered from these rheumatological disorders may not have survived to this day (e.g. the ancient Egyptian blind harpist’s, Ambroise Paré’s, Canon Van der Paele’s, Francesco Giamberti’s and those of other less well-known historical individuals) [2–4].

While their diagnosis can be considered possible, we wish to stress how greater methodological caution should be implemented [5, 6] when proclaiming that this represents ‘a more reliable diagnosis’ or when underlining that in Van der Paele’s mediaeval case only ‘general contemporary descriptions’ are available. Unlike Antinori’s case, all the previously proposed cases present morphological evidence of temporal arterial changes.

On examining the patient, the skilled physician Venanzio Lupacchini (1730–1775) recorded, as the authors summarized, several signs and symptoms, which they suggest fit the criteria for PMR and GCA [1]. However, no mention of the morphology of the temporal region, which often presents reddening and desquamating as a result of the process, is recorded by Lupacchini as reported by the authors themselves [1, 7]. Finally, the reference to ‘arms palsy’ should have been given in its original language (i.e. not only in translation) and contextualized in the medical terminology and theory of that time, a view justly supported by one of the two authors (A.F.) [8]. Moreover, the approach used in their paper seems to be at variance with author L.V.’s statement alibi that ‘paleopathology should not be intended as a game of words … but an evidence-based discipline’ [9]. For this very reason, the authors’ interpretation of the arms palsy is the weakest part of their argument and so reduces the sensitivity of their analysis.

Having said that, Antinori’s symptoms and this study add to the body of literature that can be used to stimulate and educate trainees in rheumatological diseases.

## Data Availability

All data supporting this article can be supplied by the authors upon reasonable request.
